# Books Reviewed

**Published:** 1869-01

**Authors:** 


### Editorial Department.

### Books Reviewed.

Atropia: Its Chemical, Physiological, and Therapeutic Action; together with experiments instituted to ascertain its toxicological properties. By Samuel R. Percy, M. D.

The author writes, first, the history of the drug, which is obtained from all parts of the belladonna-plant, but in largest quantity from the root. He then describes the process for obtaining atropia and its salts, and refers to the various sources of information upon this point, proceeding to a description of its physical properties and its behavior with chemical tests and reagents. He then describes the physiological action of atropia upon animals and relates some very interesting experiments showing its effects when introduced into the stomach, and when injected under the skin, and also when injected into a vein. The antagonistic effects of opium and belladonna when administered to animals is illustrated by experiments. The physiological action of atropia on man is shown by detail of facts and experiments. We make the following quotation upon the antagonistic action of opium and belladonna:

"Prof. A. von Graëfe makes the following observations on the antagonistic action of opium and belladonna injected into the cellular tissue: When a solution of atropine has been injected hypodermatically, three or four minutes afterwards the pupil becomes dilated, the pulse rises to 140–160, and other symptoms of narcosis by atropine are observed. If morphia is then injected, all these phenomena, which would otherwise last for hours, disappear in a very short time. After a hypodermic injection of morphia, a considerable myosis is observed, and the pupil cannot be dilated. This is probably to be ascribed to an active irritation in the sphincter muscle, just as mydriasis caused by belladonna is to be explained by active irritation of the dilatator muscle. A new fact, which Von Graëfe has observed, is the antagonistic action of these medicines upon the faculty of accommodation; although it has not occurred in all the cases in which he has operated. *Atropine causes paralysis, and morphia a spasm of accommodation.* In consequence of this, the space allowed to accommodation becomes greatly limited, and myopia is the result. All distant objects are indistinctly seen; but, if concave glasses are used, this is obviated. It is true that the myopia is not so considerable as it appears to be when trials on both eyes are made, as, if only one eye is experimented upon, distant objects are more clearly distinguished; a circumstance which is, no doubt, due to the weakening action of morphia upon the internal muscles of the eye. But the phenomenon is only temporary, and is generally only observed three-quarters of an hour after the injection. It is probable that, if a stronger dose of morphia were used, it would last longer and also be more constant; but it would not be justifiable to do this in order to satisfy physiological curiosity. The symptoms described are to be explained in the following manner: Opium and belladonna have an antagonistic effect upon the muscular fibres of the tensor chorioidæe, as upon the muscles of the iris; and the analogy would be quite complete, if a double and antagonistic innervation of the tensor chorioideæ, by both the third pair and the sympathetic nerve, was just as certain as it is for the iris."

Of the therapeutics of atropia, we will also quote briefly our author:

"A very large number of persons are troubled with a passive constipation of the bowels. This is especially the case with many females of delicate health and highly nervous sensibility. With such persons, I have in many instances given permanent relief by the careful use of the sulphate of atropia. * * * *

"Atropia has been highly recommended in the treatment of various other diseases, as in asthma, in rheumatism, in whooping-cough, in scarlet fever, in spasmodic stricture of the urethra, in strangulated hernia, in rigidity of the os uteri, etc.; but these disorders are more easily cured by other remedies that are less dangerous in their use.

"Atropia, as well as belladonna, has been very highly vaunted *as a prophylactic against scarlet fever.* I have tried it many times for this purpose, but all my success, if it can be called success, has been purely of a negative character, and, in many instances where I have used it, it has had no effect in preventing the disease. Children bear a larger dose than adults, and the effects pass off more rapidly.

"To the ophthalmologist, atropia, or its congener, daturia, is indispensable.

"'Hahnemann and his followers have made the assertion that the administration of belladonna produces a rash similar in appearance to scarlatina, and, upon this assertion, they use belladonna as a cure for scarlet fever. Many eminent medical men accept this assertion as a fact, and, in one of our latest and best works on 'Skin Diseases,' the author, in enumerating articles that produce an erythema, states the reïterated assertion that 'belladonna produces a rash of a rosy hue.' Dr. Fuller, in a paper on the 'Action of Belladonna,' with a view to solve this question, gave belladonna to a large number of patients for some months, 'in doses varying from a quarter of a grain of the extract up to seventy grains daily.' The patients were examined four or five times daily, and the occurrence of any rash or eruption was carefully looked for; but in all these cases no rash or eruption was perceived.'

"I have watched a large number of patients, both in hospital and in prlvate practice, where belladonna or atropia has been used with a like negative result. There are many unfounded assertions on the actions of medicines, but probably none so wide-spread and utterly groundless as this. It is well worthy to be classed with the other 'facts' of Hahnemannic 'provings.'"

Lastly a very full description of the poisonous effects of atropia in overdoses is shown, which we regret our inability to republish.

The essay taken collectively is one of the most interesting and instructive medical papers of the season. The antagonistic properties of opium and belladonna are attracting the attention of observers, and numerous experimenters are now trying to determine the questions involved. The author has contributed a most valuable and suggestive paper upon the subject, which will be regarded with deep interest by all. His experiments will be repeated and varied, and we have no doubt his conclusions will be sustained.

---

Annual Report of the Commissioner of Agriculture, Hon. Horace Capron, for the year 1868.—We extract the two following paragraphs from this report, as of especial interest to physicians. We have nothing additional to suggest upon either subject, since it has been presented in most full and impressive manner by the Commissioner. Every member of the medical profession will at once appreciate the value and importance of this report. Nothing could be better expressed, or better directed:

*Diseases of Farm Stock.*—The prevalence of fatal maladies among all varieties of farm animals, resulting in the annual loss of not less than $50,000,000, demands the prompt attention of this Department, the vigilance of the agricultural associations, and National and State legislation. The past year has not been one of peculiar misfortune in this respect, except in the dissemination of the splenic fever, communicated by Texas cattle; yet horses, mules, sheep and swine, have all suffered from the local prevalence of malignant forms of disease, against which little veterinary skill is opposed, and little more than empiricism and superstitious folly is practiced. A disease may suddenly decimate the cattle or horses of a neighborhood, the only popular knowledge of which is the statement that it is a murrain or distemper. A disease exists locally in several of the Southern States, by which the total loss of a plantation's stock of horses and mules not unfrequently occurs, with scarcely an effort or hope for a cure. The annual losses in swine cannot be less than $10,000,000 or $15,000,000 by the disease commonly known as "hog cholera," for which no remedy has been found; and prevention has proved difficult and uncertain.

On the breaking out of the splenic fever at the halting places of Texas cattle during the past summer, I commissioned Prof. John Gamgee, of the Albert Veterinary College of London, to investigate its character and causes and the means for its prevention. The labor was undertaken at once and continued with zeal and activity in several Western States, including the Texas cattle stations of western Kansas. Post mortem examinations, not only of diseased native stock but of the cattle from Texas, were repeatedly made, and their results carefully recorded, all tending to connect the migrating herds of the Gulf coast unmistakably with the existence and spread of the disease. The report of this investigation, enriched with valuable material collected by the statistical division of this department for a history of the outbreak, will be presented to Congress at an early day, together with a statement of the previous history of this disease in this country, and chromo-lithographs of internal organs of animals dying from the disease. The department has been cramped for means to conduct this investigation, having no fund from which to defray its expenses, except that for statistical purposes, which is quite too meagre for the absolutely indispensable demands upon it, and congressional aid will therefore be requisite for the completion of the work undertaken and for the proper publication of the report upon it.

While it is deemed important to investigate the cattle diseases prevalent, and to obtain the best professional aid in seeking to diminish the extent of their ravages, it is evident that effort directed toward the cure of any disease which is well developed in any section of the country must be very unsatisfactory and ineffectual. Many of the diseases of cattle, as of men, have their origin and distribution in the unnatural and unhealthy conditions of their growth and management, naturally resulting from what is termed our civilization. These diseases belong to the class of ailments which are preventible. Their causes are known, and means of prevention are at our disposal; and if an enlighted state of public opinion leads to the formation of societies for the prevention of cruelty to animals, a higher appreciation of the dependence of domestic animals upon us, not only for food but for care and protection from disease, should lead to the formation of establishments for the study of cattle in health and disease, and the training of a class of practitioners who would bring the highest medical skill to the treatment of our domestic animals. If motives of humanity should fail to influence, self-interest, in view of the annual losses of millions of dollars in valuable property, should be a potential inducement to prompt action in this direction. The formation of veterinary colleges—not for the treatment of animals, but for the education of a class of practitioners of skill and science, who might become beacons, warning the proprietors of stock of the approach of disease, and pointing out the means of prevention—has been adopted in many European States, from which much benefit to the community has been derived. I consider it eminently the duty of this department not only to point out the want of such an institution but to initiate its establishment; and I earnestly hope that Congress may authorize at an early day the creation of a division of veterinary surgery for the investigation and prevention of diseases of domestic animals, and for the advancement and diffusion of veterinary science and for its most efficient and beneficent practical operation.

*Cinchona Planting.*—Among the "new and valuable plants" which the organic law of the department requires it to propagate, cultivate, and distribute among agriculturists, there may be included not merely those useful as food stuffs, or for industrial arts and manufactures, but also those which subserve the sanitary interests of the people. European governments, possessing intertropical colonies, have already taken the lead in the introduction and acclimatization of medicinal plants within their own limits. I would especially call attention to the necessity which has arisen within the last few years for the initiation of prompt measures by the government to obviate the results of the extinction of the cinchona forests on the Andes, which is caused by the negligence of the governments of Peru, Ecuador, and more northern Andean States. The experiments of England, Holland, and other countries, have shown how readily new plantations of cinchona trees may be established in suitable localities, how rapidly the species becomes acclimated, and how early it yields satisfactory returns, and how easily such enterprises are popularized and rendered profitable. The supply of quinine has become a necessity of existence, not merely as a cure, but as a prophylactic agent. During the late war many thousand lives were saved by its use alone. In view of the approaching extinction of the cinchona species, (unless intelligent governments introduce the cultivation within their own territories,) I would earnestly recommend that an appropriation be made by Congress to introduce it, and to propagate and establish a cinchona plantation under the care of this department. The attention of the public has already been called to this subject in the annual report for 1866, and the present is a fitting time for carrying into effect the plan there recommended.

---

The Physician's Medical Compend and Pharmaceutical Formulæ, compiled by Dr. Edward H. Hance. Hance, Griffith & Co., Philadelphia, 1868.

This is Materia Medica and Therapeutics, condensed into a very small pocket-book, and Poisons and their antidotes, tables of nearly all sorts, directions for writing prescriptions, abbreviations commonly used, and a great amount of other similar material added. It makes a pocket companion for the young physician which might greatly aid him in early years of practice. It is conveniently arranged and beautifully bound, and we have no doubt will prove a great favorite with many—with all, who require a condensed guide in the preparation and dispensing of medicine.

---

Physician's Daily Pocket Record. By S. W. Butler, M. D.

We are indebted to Dr. S. W. Butler of the Philadelphia Medical and Surgical Reporter, for the most elegantly bound, and in every way the most attractive "Pocket Record" for physicians we have yet seen. It contains all the solid attractions which are ever embodied in these books, and adds to them an elegance not elsewhere observed.

The Materia Medica in its Scientific Relations. Published by Judd & White, New Haven, Conn.

We have received a very carefully written, thoughtful and philosophical paper with the above title, but the author's name does not appear. "*How can the Materia Medica be built up as a Science of Observation?*" is the question discussed. The subject is treated in a masterly and suggestive manner, and we think the author has done himself credit as a careful thinker and sound reasoner. We suggest that he issue a second edition of his pamphlet and let himself be known.

---

The Present Problems in Abdominal Section; illustrated by a successful case of double Ovariotomy, by Prof. Horatio R. Storer, M. D., of Boston, Mass.

This paper contains a brief discussion of many points in abdominal section, and some suggestions in modes of treatment which the author regards as diminishing the present small mortality from these operations. It also contains a full description of a case of successful removal of both ovaries.

---

Correlation of the Physical and Vital Forces. Inaugural Address, introductory to the Course on Institutes of Medicine in the Jefferson Medical College. By J. Aitken Meigs, M. D.

This is a discourse upon "certain historical points connected with the great doctrine of the correlation of the Physical and Vital Forces, at present so strongly attracting the attention of the scientific world." The author adopts the following sentiment, or, at least, quotes it from Dr. Metcalfe, as embodying one of the historical points upon which he discourses: "So long as the present doctrine of a vital force in nature prevails, so long will this 'riddle of death' remain unsolved, so long will we stand in the presence of this sphynx, confounded and amazed, so long will the phenomena of life, disease and death remain for the physiologist, the pathologist and physician, a series of facts without co-ordination and without harmony."

He goes on to show that in the latter part of the eighteenth and first half of the present century, physiologists, with but few exceptions, contended for the existence of a vital or peculiar governing principle or power in organic beings as the fundamental cause of growth, nutrition and all the other phenomena of life.

The first to announce an opposite view was Dr. S. L. Metcalfe, who, in his work upon "Caloric," maintained the existence of a substantive vital principle, not dis tinct from all other things in nature, but, on the contrary, identical with the *essentia caloris*, or, subtle fiery ether, which Pythagoras regarded as the principle of life, animating the whole system of nature. He says that, "with wonderful precision and skill, Dr. Metcalfe heaped fact upon fact, to prove that caloric intimately combined with, and acting through, organic matter as a peculiar medium, is the cause of all the complex phenomena of life; is in fact converted into the vital principle.

This address cannot be abbreviated or condensed, and any adequate idea conveyed of its scope and design; it is to be carefully read that its numerous suggestions may be comprehended. It embodies the many interesting questions now agitated upon the ultimate cause or source of life. and will be perused with the deepest interest by those who have taste and relish for theoretical and speculative truth.

